# Complex Interaction of Deferasirox and *Pythium insidiosum*: Iron-Dependent Attenuation of Growth *In Vitro* and Immunotherapy-Like Enhancement of Immune Responses *In Vivo*

**DOI:** 10.1371/journal.pone.0118932

**Published:** 2015-03-04

**Authors:** Régis A. Zanette, Paula E. R. Bitencourt, Dimitrios P. Kontoyiannis, Rafael A. Fighera, Mariana M. Flores, Glaucia D. Kommers, Priscila S. Silva, Aline Ludwig, Maria B. Moretto, Sydney H. Alves, Janio M. Santurio

**Affiliations:** 1 Graduate Program in Pharmacology, Health Science Center, Federal University of Santa Maria (UFSM), Santa Maria, RS, Brazil; 2 Graduate Program in Pharmaceutical Sciences, Health Science Center, UFSM, Santa Maria, RS, Brazil; 3 Department of Infectious Diseases, Infection Control and Employee Health, The University of Texas M. D. Anderson Cancer Center, Houston, Texas, United States of America; 4 Graduate Program in Veterinary Medicine, Health Science Center, UFSM, Santa Maria, RS, Brazil; Public Health Research Institute at RBHS, UNITED STATES

## Abstract

*Pythium insidiosum* iron acquisition mechanisms are unknown. We previously showed that the iron chelator deferasirox had weak activity *in vitro* and in rabbits with experimental pythiosis. Here we show that deferasirox causes damage to *P*. *insidiosum* hyphae *in vitro*, but that activity is diminished in the presence of exogenous iron. The tissue activity of the proinflammatory enzyme adenosine deaminase and the histological pattern observed in pythiosis lesions of rabbits treated with deferasirox were similar to the ones in animals treated with immunotherapy.

## Introduction

Infections by the oomycete *Pythium insidiosum* often cause cutaneous, gastrointestinal, ocular or systemic disease in humans and animals. Despite significant advances in diagnosis and treatment, pythiosis remains a difficult-to-treat infection, characterized by necrotizing eosinophilic and granulomatous inflammation and frequent lack of response to antifungal therapy [1, 2].

It is known that the purinergic system participates in immune system modulation during infections, including in pythiosis [3]. In particular, an enhanced adenosine production results from the ATP released into the extracellular space by injured or pathogen-stimulated cells, thus limiting hyperergic inflammatory responses [3]. In turn, the enzyme adenosine deaminase (ADA) represents a crucial immunomodulatory mechanism aimed at counteracting excessive extracellular adenosine production and avoiding excessive immunopathology [4, 5]. Moreover, ADA has been found to participate in the regulation of several immune cell types, including macrophages, lymphocytes, neutrophils and dendritic cells [4, 6].

Iron is essential for both the pathogen and the host, and not only iron deficiency but iron overload can significantly impair immune function by altering T and B cell proliferation [7]. The predisposition of thalassemic, iron overloaded patients to pythiosis, the observation of iron deficiency anemia in rabbits experimentally infected with *P*. *insidiosum* [8] and the report of a functional ferrochelatase gene in *P*. *insidiosum* has elicited interest in the role of iron in pythiosis [9].

The iron chelator deferasirox has shown potential as a therapeutic intervention for pathogenic fungi [10, 11]. Therefore, it was hypothesized that this drug would be of interest in patients with pythiosis in view of its iron chelating and immunomodulatory properties [2, 11]. Nevertheless, deferasirox showed only a weak activity *in vitro* against *P*. *insidiosum* strains. Similarly, subcutaneous lesions were only slightly decreased following deferasirox treatment when compared to placebo-treated animals [8]. To better understand the effect of deferasirox on *P*. *insidiosum* growth and to explore the immunomodulatory properties of deferasirox in pythiosis lesions, we evaluated the activity of deferasirox *in vitro* and in lesions of rabbits treated with the iron chelator and/or immunotherapy.

## Materials and Methods

### *P*. *insidiosum* growth rate

We determined the time course of deferasirox-induced hyphal damage with the use of the 2, 3-bis(2-methoxy-4-nitro-5-sulfophenyl)-2*H*-tetrazolium-5-carboxanilide (XTT) colorimetric assay. Triplicate Eppendorf tubes containing 10^3^ *P*. *insidiosum* zoospores (strain CBS 101555) diluted in 1 mL RPMI 1640 broth and deferasirox at the MIC concentration (50 μg/ml) [8], iron (0.125% FeCl) [10], or both, were incubated at 37°C on a rotary shaker. Drug-free RPMI 1640 was used as the control medium. At the 0-, 4-, 8-, 12-, 16-, 20- and 24-h time points, tubes were removed from incubation and the XTT (Sigma) assay was performed as described previously [12]. The optical density curves were measured at 405 nm with use of a plate reader (PowerWave HT; BioTek). Wells without zoospores served as blank controls.

### DiBAC viability staining

To determine whether deferasirox causes damage to *P*. *insidiosum* hyphae, we performed viability staining with bis-(1, 3-dibutylbarbituric acid) trimethine oxonol (DiBAC) (Molecular Probes, Carlsbad, CA). *P*. *insidiosum* zoospores were incubated in Eppendorf tubes with RPMI 1640 plus 0.15% (wt/vol) Junlon (Nihon Junyaku, Tokyo, Japan) at 37°C with shaking for 4 h. Tubes were centrifuged at 4,000 g to remove the media, and germlings were resuspended in RPMI containing deferasirox at the MIC concentration, 0.125% iron or both. Drug-free medium served as negative control. After 6 h incubation at 37°C with shaking, DiBAC staining was performed as previously described [12]. After the final wash, the samples were immediately suspended for fluorescent microscopy at room temperature.

### Animal procedures

Rabbit pythiosis lesions used in the current study for histopathology and ADA activity assessment were obtained from a previous study in which groups of five *P*. *insidiosum* infected animals were treated by oral gavage with 15 mg/kg/day deferasirox, subcutaneous immunotherapy (PitiumVac, LAPEMI/EMBRAPA, Brazil) at 14 d intervals, a combination of both, or placebo, with treatments starting 25 d post-infection [8]. At day 75, animals were anesthetized and euthanized following procedures approved by the Animal Welfare Committee of the Federal University of Santa Maria. The subcutaneous lesions were measured using a slide caliper, removed, rinsed in deionized water and 1-cm thick slices were sampled away from the edge of the lesions. Samples for histopathological analysis were fixed in 10% buffered formalin, whereas those for the enzymatic assay were stored at -80°C.

### Histopathological evaluation

Lesion samples were fixed in formalin, embedded in paraffin and sections stained with hematoxylin and eosin (H&E) to evaluate the inflammatory cell infiltrate and Grocott’s methenamine silver nitrate (GMS) to evaluate hyphal morphology [13]. Two lesion samples were evaluated per animal, and two histological sections were performed in each slice. A representative field was photographed at 40x magnification.

### Enzymatic assays

Lesion slices were washed and homogenized in (1:10, w/v) 50 mM phosphate buffer solution (pH 7) on ice using a tissue homogenizer. The homogenate was centrifuged at 10,000 X g for 60 min and clear upper supernatant fluids were used for protein and ADA activity measurements. Protein was determined by the method of Lowry et al. [14], and ADA activity was estimated spectrophotometrically as described by Giusti [15], using 75 μM ammonium sulphate as ammonium standard. The results were expressed as U/L/mg of protein.

### Statistical analysis

The variables were compared among groups by one-way analysis of variance and Tukey’s test (α = 0.05). Furthermore, a Pearson’s correlation analysis was performed to evaluate the relationship between mean lesion areas and enzymatic activities.

## Results

### Iron availability is essential for *P*. *insidiosum* growth

**Fig 1:**
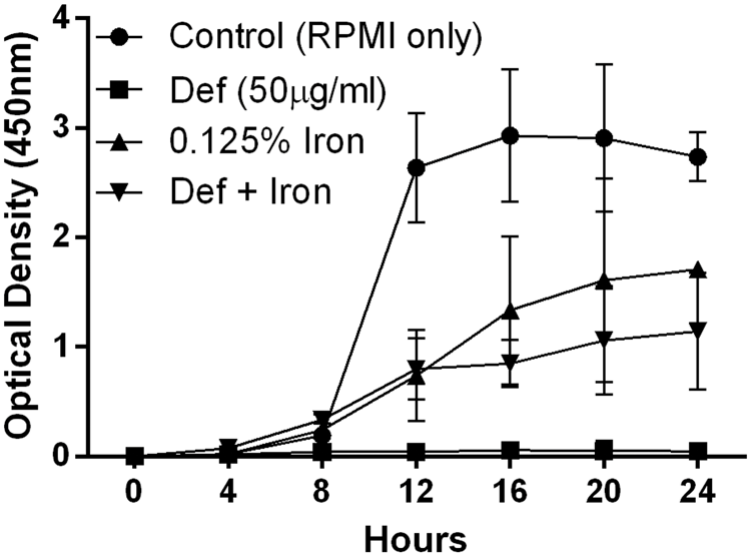
XTT reduction assay. *P*. *insidiosum* zoospores were grown in RPMI 1640 in the presence of deferasirox (50 μg/ml), 0.125% iron (FeCl_3_) or both. No growth was observed in the presence of deferasirox at the MIC concentration (50 μg/ml). The addition of iron abrogated the inhibitory effect of deferasirox, but the treatment with FeCl_3_ alone did not promote growth to levels compared to control (RPMI only). Values represent the means ± SEM of three separate experiments.

*P*. *insidiosum* zoospores in RPMI 1640 medium reached a peak of growth at 16 h of incubation. At the MIC concentration, the iron chelator deferasirox fully inhibited *P*. *insidiosum* growth, and this inhibition was partially abrogated by the concomitant addition of 0.125% FeCl_3_ (Fig. 1). Interestingly, the growth rate of *P*. *insidiosum* supplemented with iron was lesser than the observed in the control medium (RPMI only).

### Deferasirox damages *P*. *insidiosum* hyphae

**Fig 2:**
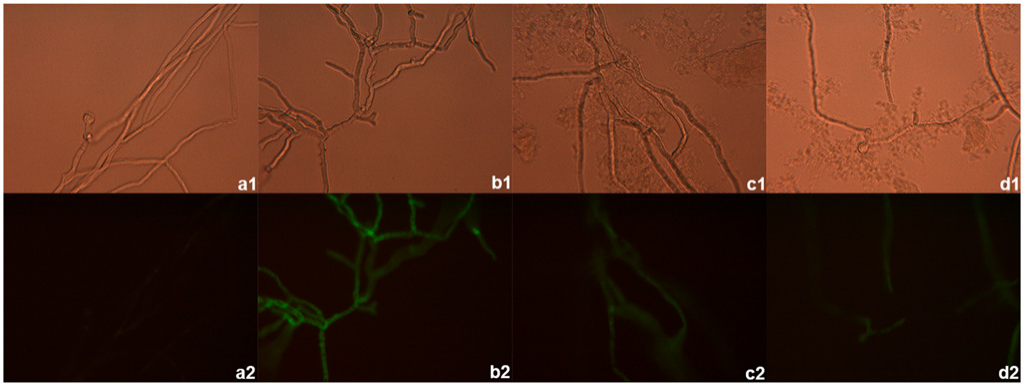
DiBAC viability stain. *P*. *insidiosum* germlings were exposed to deferasirox at the MIC (50 μg/ml) (b1, b2), 0.125% iron (c1, c2) or both (d1, d2) for 6h. Untreated germlings were used as negative controls (a1, a2). Mycelia subsequently were stained with DiBAC and observed with bright-field (a1 to d1) and fluorescent microscopy (a2 to d2). The fluorescence of the dark pictures is indicative of hyphal damage.

The DiBAC staining showed enhanced fluorescence, indicating fungicidal activity, mainly in the subapical compartments of *P*. *insidiosum* germlings treated with deferasirox at the MIC concentration (Fig. 2b), when compared to untreated controls (Fig. 2a). Increased DiBAC uptake was also observed in the group treated with deferasirox and iron (Fig. 2c), and in a lesser degree in the group treated only with iron (Fig. 2d).

### Deferasirox treatment mimics immunotherapy in rabbits with experimental pythiosis

**Fig 3:**
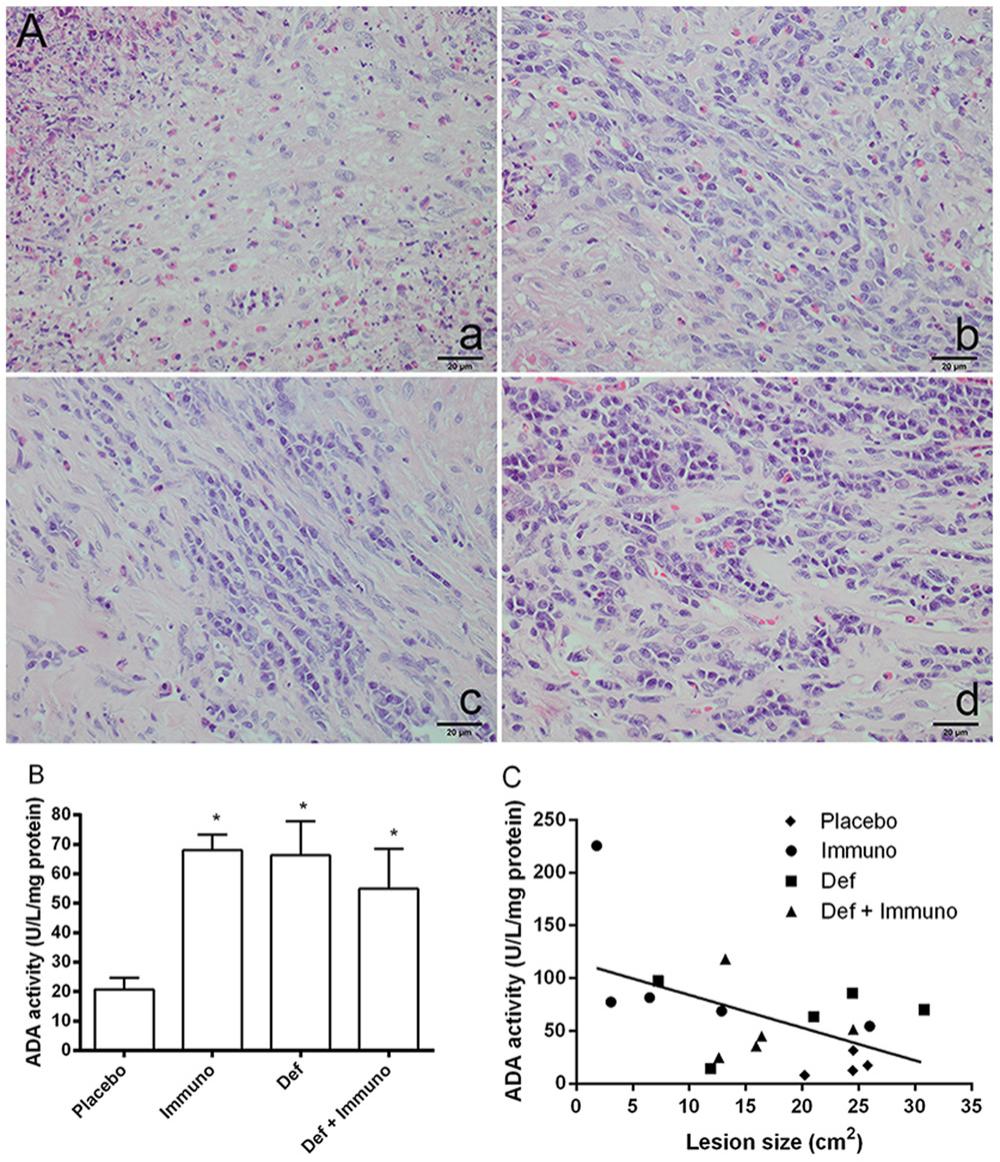
Histology and enzymatic activity of rabbit pythiosis lesions. **A**) Representative histological sections of rabbit pythiosis lesions (H&E stain, 40x magnification). a) Placebo, area of necrosis and fibroplasia with eosinophilic infiltrate and rare lymphocytes and plasma cells; b) immunotherapy, area of fibroplasia with increased number of lymphocytes and plasma cells and moderate quantities of eosinophils and macrophages; c) deferasirox, area of fibroplasia mainly composed of lymphoplasmacytic infiltrate and rare eosinophils; d) deferasirox and immunotherapy, area of fibroplasia with prominent lymphoplasmacytic infiltrate and rare eosinophils. **B**) Expression of ADA in rabbit pythiosis lesions. ADA activity was significantly increased in all treated groups when compared to placebo (*P = 0.03). Values are shown as means ± SEM. **C**) A negative correlation was observed between lesion ADA activity and lesion size (Pearson *r* = -0.53; *P* = 0.02).

The impact of deferasirox in modulating the immune response was compared to immunotherapy by histopathological analysis and by measuring ADA activity in the lesions of rabbits with experimental pythiosis. Lesions in the placebo group showed eosinophilic necrosis surrounded by macrophages, epithelioid cells, multinucleated giant cells and a few plasma cells and lymphocytes. In contrast, cellular infiltrates mainly composed of plasma cells and lymphocytes were observed in H&E stained lesions for all treated groups (Fig. 3A). The histological data are concordant with the increase observed in lesion ADA activity in deferasirox and/or immunotherapy-treated groups when compared to placebo (*P*<0.05; Fig. 3B). Moreover, a negative correlation between ADA activity and mean lesion sizes was observed in our study (Fig. 3C).

## Discussion

In assessing the effect of iron on *P*. *insidiosum* growth *in vitro*, it was noted that the use of the iron chelator deferasirox fully abrogated hyphal growth. The green fluorescence observed mainly in hyphae treated with deferasirox, which was partially abrogated by the addition of iron, indicates that the cidality of deferasirox was mediated through iron deprivation [10, 11]. Notwithstanding, enhanced DiBAC uptake was also observed in the group treated only with iron, suggesting that 0.125% FeCl_3_ could have been toxic for *P*. *insidiosum in vitro*. In line with this, the addition of exogenous iron to growth medium has been reported to not enhance fungal growth, probably because trace levels of iron in RPMI medium allow fungi to grow very well [16]. The use of fluorescent dyes such as the safranine and the acriflavin would add additional knowledge in iron-specific changes in cell wall and organelle of *P*. *insidiosum* [17].

Iron overload is known to exacerbate fungal infections such as aspergillosis, candidiasis and especially mucormycosis by enhancing aggressive proliferation of microbes in host tissue [11, 16, 18]. The elevated iron availability also impairs host immune responses, as observed in patients with thalassemia or hemochromatosis, which frequently have reduced CD8+ T-cell counts that respond positively to iron chelation therapy [7]. Current evidence shows that the mechanisms of cure of the immunotherapeutic treatment against pythiosis are based on the switch from a Th2 (eosinophils) to a Th1 (macrophages and lymphocytes) subset [3]. Therefore, the impact of deferasirox in modulating the immune response was compared to immunotherapy by histopathological analysis and by measuring ADA activity in the lesions of rabbits with experimental pythiosis. Microscopical evaluation of the lesions demonstrated that the immunomodulatory mechanisms of the iron chelator were reminiscent to the changes following the immunotherapy. Interestingly, the type of inflammatory response did not affect the morphology of *P*. *insidiosum* hyphae, only the number of hyphae in each group (GMS stain; S1 Fig.), in agreement with a previous study showing that differences are only numeric [19]. Conversely, Martins et al. [13] showed that hyphal morphology (integrity) is normal when amongst eosinophils (necro-eosinophilic pattern) and disintegrated when associated with giant cells (granulomatous pattern). We speculate that, although the immunomodulatory properties of deferasirox were able to change the pattern of the cellular infiltrates observed in the treated groups, other factors such as poor bioavailability of the drug in the granulomatous lesions and different iron sources might not have reproduced the hyphal damage observed *in vitro*. A pioneer report on the use of deferasirox as salvage therapy in a human patient with pythiosis, although unsuccessful, mentioned a degree of symptom relief with the iron chelation therapy [20]. Moreover, rabbits with pythiosis treated with deferasirox showed increased liver iron content in comparison with those treated with placebo [8]. These findings corroborate our *in vitro* results showing that deferasirox is able to prevent *P*. *insidiosum* iron uptake pathways.

The increase in ADA activity observed in the treated groups emphasizes the critical role played by ADA in lymphocyte proliferation and in Th2/Th1 cytokine production and differentiation [4]. By inhibiting the proliferation of T cells and the secretion of cytokines, tissue adenosine accumulation exerts potent anti-inflammatory and immunosuppressive actions locally. Therefore, the treatment with deferasirox and/or immunotherapy neutralized the modulating actions of adenosine, as also observed by the negative correlation between ADA activity and mean lesion sizes in our study (Fig. 3C). In support to our findings, a decreased lymphocyte ecto-ADA activity has been previously reported in rabbits with pythiosis, an effect that was also reversed by immunotherapy [3]. Our data also extend previous observations showing that 1) deferasirox treatment enhanced the host inflammatory response to mucormycosis [11], and 2) ADA therapy is able to increase the secretion of Th-1 pro-inflammatory cytokines, enhancing allogeneic T-cell proliferation and immunogenicity [6]. In fact, the results of our study represent a suitable basis for further strategies using ADA therapy in animal models of pythiosis. We must emphasize that phenotypic and genotypic differences exist among *P*. *insidiosum* strains. The strain used in our study is clustered in the American group, genetically differing from the Asian isolates which commonly affect thalassemic patients [21].

In conclusion, we show that deferasirox activity against *P*. *insidiosum* hyphae *in vitro* is hampered by the addition of exogenous iron. The activity of the immunomodulatory enzyme ADA and the cellular pattern in rabbit pythiosis lesions that were treated with deferasirox were similar to those treated with immunotherapy. Such finding highlights the deferasirox-induced enhancement of the immune response.

## Supporting Information

S1 Fig Grocott’s methenamine silver nitrate staining of rabbit pythiosis lesions. No difference in hyphal morphology was observed among the groups.
(TIF)
