# Supplementary figures and images for: Complex Interaction of Deferasirox and Pythium insidiosum: Iron-Dependent Attenuation of Growth In Vitro and Immunotherapy-Like Enhancement of Immune Responses In Vivo
Source: PLoS One. 2015 Mar 4;10(3):e0118932. doi: 10.1371/journal.pone.0118932 (PMC4349436; doi:10.1371/journal.pone.0118932)

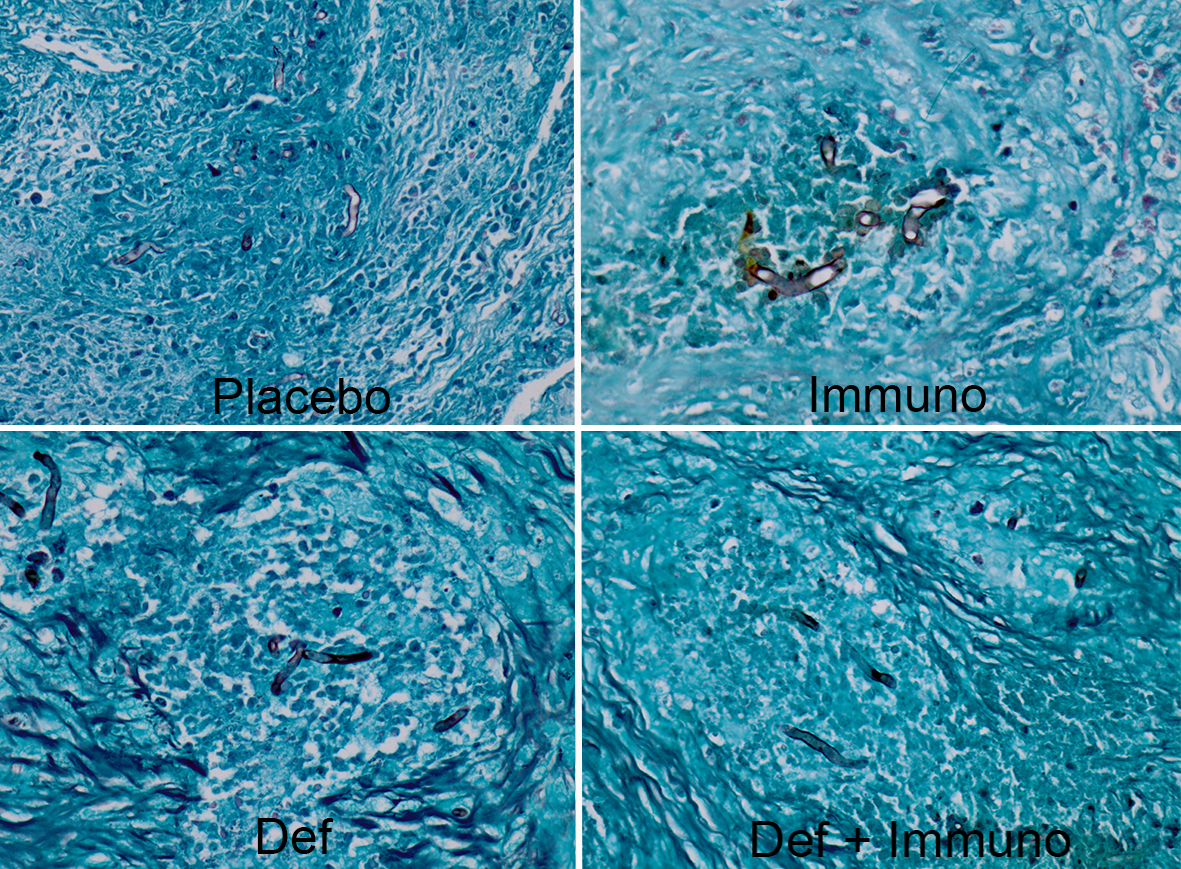

Supplement: S1 Fig — No difference in hyphal morphology was observed among the groups. (TIF) [file pone.0118932.s001.tif]
